# Bryostatin activates HIV-1 latent expression in human astrocytes through a PKC and NF-ĸB-dependent mechanism

**DOI:** 10.1038/srep12442

**Published:** 2015-07-22

**Authors:** Laura Díaz, Marta Martínez-Bonet, Javier Sánchez, Alejandra Fernández-Pineda, José Luis Jiménez, Eduardo Muñoz, Santiago Moreno, Susana Álvarez, Mª Ángeles Muñoz-Fernández

**Affiliations:** 1 Laboratorio InmunoBiología Molecular, Plataforma de Laboratorio, Hospital General Universitario Gregorio Marañón; Instituto de Investigación Sanitaria Gregorio Marañón, Madrid; Networking Research Center on Bioengineering, Biomaterials and Nanomedicine (CIBER-BBN); 2 Department of Cell Biology, Physiology and Immunology, Instituto Maimónides de Investigaciones Biomédicas de Córdoba (IMIBIC)/Reina Sofia University Hospital/University of Córdoba, Córdoba, Spain; 3 Departamento de Enfermedades Infecciosas, Hospital Universitario Ramón y Cajal, and IRYCIS.

## Abstract

Multiple studies have shown that HIV-1 patients may develop virus reservoirs that impede eradication; these reservoirs include the central nervous system (CNS). Despite an undetectable viral load in patients treated with potent antiretrovirals, current therapy is unable to purge the virus from these latent reservoirs. To broaden the inhibitory range and effectiveness of current antiretrovirals, the potential of bryostatin was investigated as a latent HIV-1 activator. We used primary astrocytes, NHA cells, and astrocytoma cells U-87. Infected cells with HIV-1_NL4.3_ were treated with bryostatin alone or in combination with different inhibitors. HIV-1 production was quantified by using ELISA. Transcriptional activity was measured using luciferase reporter gene assays by using lipofectin. We performed cotransfection experiments of the LTR promoter with the active NF-κB member p65/relA. To confirm the NF-κB role, Western blot and confocal microscopy were performed. Bryostatin reactivates latent viral infection in the NHA and U87 cells via activation of protein kinase C (PKC)-alpha and -delta, because the PKC inhibitors rottlerin and GF109203X abrogated the bryostatin effect. No alteration in cell proliferation was found. Moreover, bryostatin strongly stimulated LTR transcription by activating the transcription factor NF-κB. Bryostatin could be a beneficial adjunct to the treatment of HIV-1 brain infection.

HIV-1 can invade cells of the central nervous system (CNS) and cause progressive combined cognitive and motor impairment in infected individuals. Within days of infection, HIV-1 can enter the CNS, where various resident cell populations serve as reservoirs for the virus^1^,^2^,^3^,^4^. Macrophage and microglial cells are the primary sources of HIV-1 replication in the CNS^5^,^6^,^7^,^8^, while astrocytes are the most abundant type of cells in the CNS and connect the cells of the brain to a complex intercellular network. Because astrocytes are critical for CNS function, they should be taken into consideration in the context of HIV-1 neuropathogenesis. Therefore, low-level virus production is a consistent feature of HIV-1 infection of cultured human astrocytic cells. Introducing virus into cultured astrocytic cells, either by exposure to infectious HIV-1 or by transfection of proviral DNA, leads to an initial transient short-term burst of virus replication that is followed by a persistent phase with or without virus production.

Strong evidence suggests compartmentalization of HIV-1 in the CNS and concern that the CNS is a pharmacologic sanctuary. CNS-specific viral variants can be demonstrated in untreated individuals and may be associated with dementia^9^,^10^. Highly active antiretroviral therapy (HAART) can powerfully suppress HIV-1 replication but does not clear the virus from infected individuals. Several attempts have been made to clear the latently HIV-1 reservoir, but these have not been successful in eliminating all latently infected cells or in preventing virus rebound upon cessation of therapy^11^,^12^,^13^. These latently-infected cells are a permanent source for virus reactivation and lead to a rebound of the viral load after interruption of HAART. Therefore, current anti-HIV-1 research efforts are increasingly focused on strategies aimed at reducing the size of these persistent reservoirs of latent HIV-1 by forcing viral gene expression. This type of strategy would allow latently infected cells to die from viral cytopathic effects or host cytolytic effector mechanisms following viral reactivation, while the antiretroviral therapy would prevent spreading of the infection by the neosynthetized virus^14^,^15^.

One of these lines of research is the identification of factors that can activate HIV-1 from latency and have the potential to be used in a clinical setting. Such factors have included histone deacetylase inhibitors (HDACi), agonistic anti-CD3 antibodies, and cytokines such as interleukin (IL)-2 and IL-7^11^,^12^,^13^,^16^,^17^. A promising lead in this context is the protein kinase C (PKC) activator bryostatin, which is a macrocyclic lactone isolated from endosymbiont γ-proteobacterial *Endobugula sertul*^18^ and has shown to have diverse biological activities. The PKC family is composed of nine genes encoding ten well-characterized full-length mammalian isozymes that play different biological roles, are regulated differently, and are classified as either conventional, novel, or atypical according to the nature of their regulatory domains^19^,^20^.

It has been described that bryostatin has anti-HIV-1 activity via various mechanisms, such as blocking the effect of stromal-cell-derived factor-1 (SDF-1), which is performed by down-regulating CXCR4 receptors^21^ or modulating CD4 and CXCR4 receptors^22^,^23^. Bryostatin also causes dephosphorylation of CDK2 which inhibits RNA polymerase-II phosphorylation^24^, thus impairing HIV-1 Tat function^25^. This molecule is believed to induce the expression of HIV-1 by activating NF-κB^26^ which is required for optimal transcription of viral mRNA from the HIV-1 long terminal repeat promoter (LTR). Bryostatin-1 has been tested in clinical trials for its anti-cancer properties^27^,^28^,^29^,^30^,^31^,^32^, and it has been also investigated in preclinical models of Alzheimer Disease^33^ because it crosses the blood-brain barrier and specifically activates brain PKC^34^. However, studies using this class of compounds in clinical HIV purging strategies have not been published.

Because a significant number of astrocytes can be infected by HIV-1 in the CNS, and bryostatin may act as an activator of HIV-1 replication in other cells^23^, we investigated whether bryoststin-1 induces HIV-1 reactivation in human astrocytes and identified the downstream factors that mediate this phenomenon.

## Results

### Profile of p24 release by infected astrocytes. Activation of HIV-1 by bryostatin

**Figure 1:**
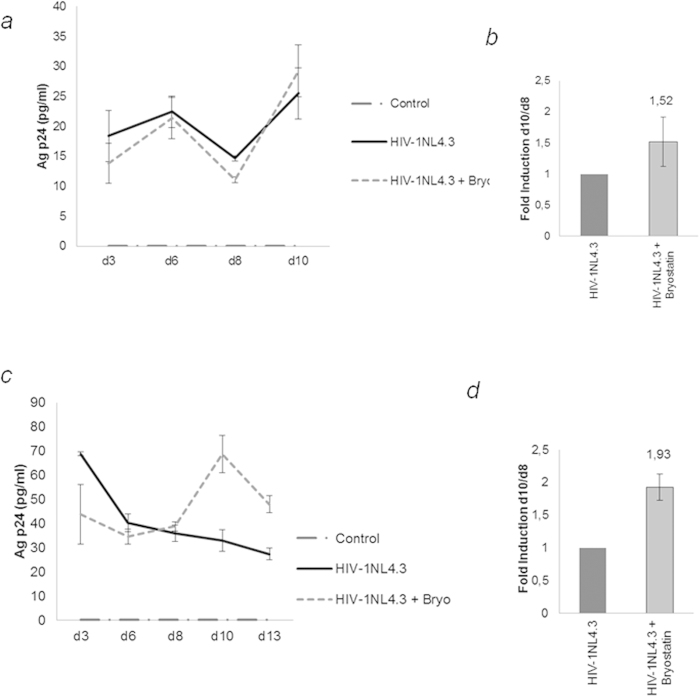
Enhancement of HIV-1 infection of latent astrocytes by bryostatin stimulation. (**a**) NHA and **(c**) U-87 cells were infected with HIV-1_NL4.3_ (100 ng p24/10^6^), and supernatants were collected at 3, 6, and 8 dpi. After collection, the cells were treated with bryostatin (100 ng/ml), and supernatants were collected 2 and 5 days later. The release of viral p24 into culture supernatants was monitored by ELISA. The fold induction of treated over infected cells 2 days after treatment is shown. The mean values (mean ± S.D.) of three independent experiments are shown. (**b**) The d10/d8 dpi ratio of NHA cells. (**d**) The d10/d8 dpi ratio of U-87 cells. The results of three independent experiments expressed as the fold increase relative to infected cells.

We first determined the optimal concentration of bryostatin in astrocytes in terms of its cellular toxicity and HIV-1 reactivation potential by measuring cell viability (Supplementary Fig. S1) and the induction of p24 production in treated cells (Fig. 1). Following HIV-1 infection, NHA primary cells were characterized for their ability to produce new HIV-1 by monitoring p24 levels in the supernatant during infection. To measure the amount of virus, cell supernatants were collected 3 days post-infection (dpi) and found to contain approximately of 20 pg/ml p24 (Fig. 1a). After 6 dpi, the level of p24 in the supernatant declined with time and was typically undetectable. After 8 dpi, bryostatin was added to the culture and its effects were analyzed 2 days later. Although NHA showed only low levels of HIV-1 infection which was consistent with other studies^1^,^2^,^35^, the production of p24 antigen was significantly up-regulated by bryostatin, which supports the levels of p24 in the supernatant of the culture that is presented in Fig. 1a. These results show direct evidence that bryostatin reactivates HIV-1 infection in NHA cells. Figure 1b represents the ratio between the p24 values at 10 dpi and 8 dpi by considering the value of infected cells without treatment as 1. Because the highest increase in replication without an effect on cell viability was observed at a dose of 100 ng/ml, this concentration was used in the subsequent experiments. Furthermore, bryostatin was able to reactivate latent HIV-1 in the U-87 cell line (Fig. 1c), and as expected, this reaction was stronger than in primary cells. We found that the effect produced by the bryostatin at 10 dpi, disappeared 5 days later (13 dpi). The d10/d8 dpi ratio is shown in Fig. 1d. It is important to note that following time-course treatment with bryostatin, reactivated HIV-1 replication was found to be transient because the levels of p24 dropped in treated cells within 5 days (13 dpi).

To confirm that the observed effect of HIV-1 production was not due to changes in cell proliferation, proliferation assays were performed in parallel with the viral activity assays in both cell types. The results consistently demonstrated minimal proliferation associated with bryostatin treatment of astrocytes up to 72 h (Supplementary Fig. S2).

### Bryostatin modulates HIV-1 latency via PKC signaling ******

We sought to investigate whether bryostatin could reactivate latent HIV-1 infection in human astrocytes via modulating PKCs and identify which specific PKC isozyme was involved in the activation loop.

**Figure 2:**
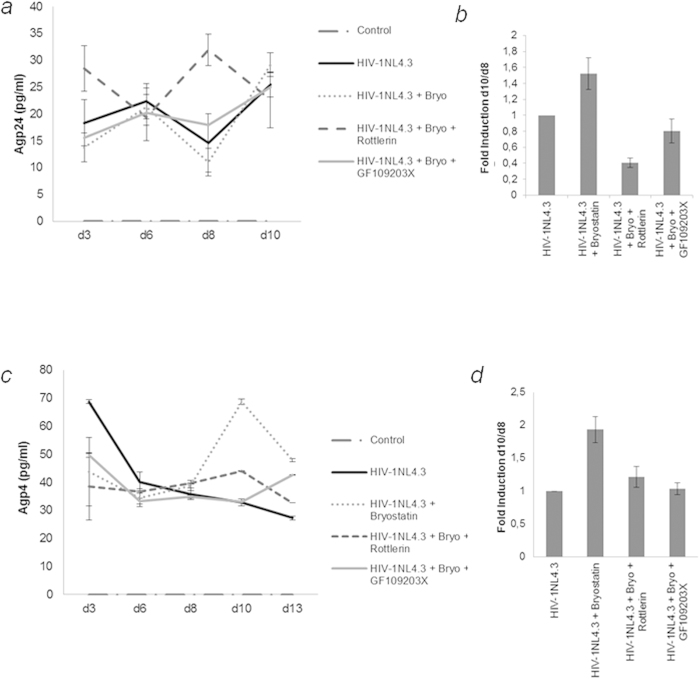
Bryostatin reactivates latent HIV-1 infection via activation of classical and novel PKCs. (**a**) NHA and (**c**) U-87 cells were pretreated with bryostatin either alone or in combination with a classical PKC inhibitor GF109203X (10 μM) or the novel PKC inhibitor rottlerin (5 μM) as indicated and monitored using ELISA. The mean values (mean ± S.D.) of three independent experiments are shown. (**b**) The d10/d8 dpi ratio of NHA cells. (**d**) The d10/d8 dpi ratio of U-87 cells. The results of three independent experiments expressed as the fold increase relative to that of infected cells.

To ascertain the role of PKC isoforms, various pharmacological inhibitors were used in combination with bryostatin. The non-selective PKC inhibitor GF109203X (10 μM) significantly inhibited the increase in viral reactivation that was induced by bryostatin, in NHA (Fig. 2a) and U-87 cells (Fig. 2c). Rottlerin (5 μM), a specific inhibitor of PKC-δ, also reduced the viral reactivation induced by bryostatin, especially in primary cells. These data reveal that bryostatin induced viral reactivation is mediated, at least in part, by conventional and novel PKC isoforms. Figure 2b,d represent the ratios between the p24 values at 10 dpi and 8 dpi in NHA and U-87 cells, respectively, considering the value of infected cells without treatment as 1.

### Bryostatin mediated-HIV-1-reactivation involves activation of NF-κB

**Figure 3:**
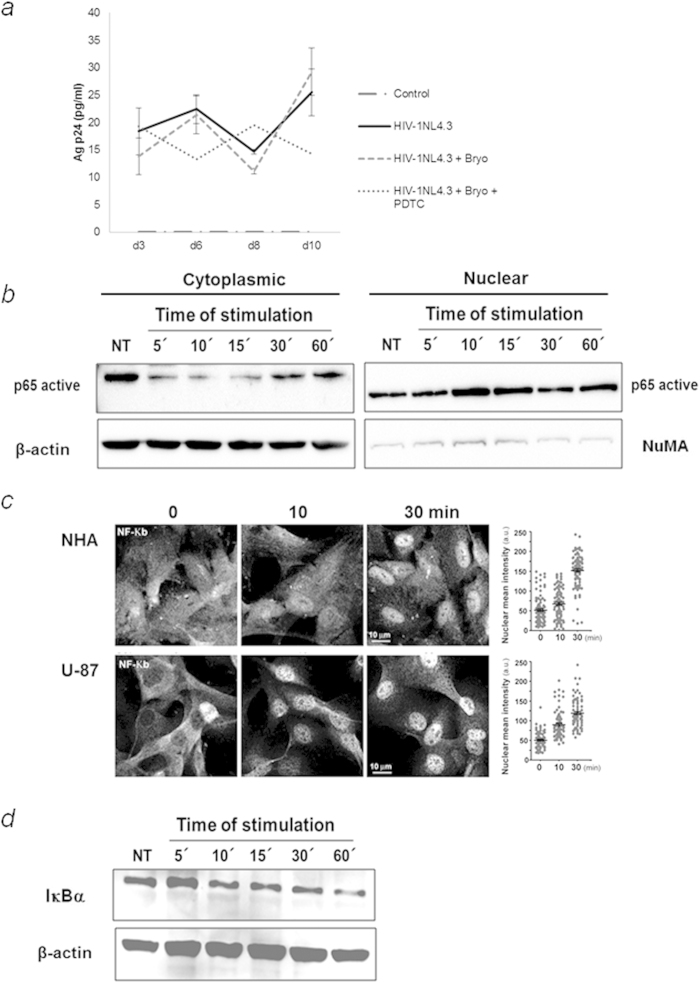
Bryostatin induces viral reactivation in a NF-κB-dependent manner. (**a**) The p24 levels were monitored 2 and 5 dpi after treatment with bryostatin alone or in combination with PDTC (10 μM) in NHA supernatants. The mean values (mean ± S.D.) of three independent experiments are shown. (**b**) NHA cells were treated with 100 ng/ml bryostatin for the indicated times. Nuclear translocation of NF-κB upon treatment was measured using Western blotting. Antibodies directed against β-actin and NuMA were used as protein loading controls for the cytoplasmic and nuclear fractions, respectively. (**c**) Confocal immunofluorescent analysis of NHA and U-87 treated with or without bryostatin (left) using Phospho-NF-κB p65 (Ser536) Rabbit mAb (green). Nuclei were labeled with TOPRO. The nuclear:cytoplasmic ratios of immunostaining were measured at the single-cell level by quantifying the NF-κB intensities inside and outside of the nucleus (blue TROPO). Data for 500 single-cell measurements are shown for cells treated with or without NHA and bryostatin (100 ng/ml) for the indicated times (100 ng/ml) (right panel). Scale bar, 10 μm. (**d**) Bryostatin induces IκBα degradation. U-87 cells were cultured with bryostatin for the indicated time points (0–60 min). The cell lysates were blotted with antibodies specific for IκBα. Western blot data are representative of 3 independent experiments.

PKCs have also been shown to activate HIV-1 transcription via NF-κB^36^. To dissect the possible involvement of this transcription factor in viral reactivation mediated by bryostatin, the effect of the agent PDTC, shown in previous work to selectively inhibit NF-κB activation^37^,^38^, when used prior to bryostatin treatment was determined. The reactivation of HIV-1 was affected by the presence of PDTC and this treatment reduced p24 levels in the cell supernatants at 10 dpi with similar efficiency as PKC inhibitors in NHA cultures (Fig. 3a). The effects of PDTC and BAY11-7082, an irreversible inhibitor of IKKα on bryostatin-mediated reactivation in U-87 cells are shown in Supplementary Fig. S3.

The activation of NF-κB usually involves degradation of the IκBα subunit bound to the NF-κB dimer, which allows its translocation to the nucleus, where it binds and activates NF-κB-dependent genes. Due to this ability of NF-κB to translocate from the cytoplasm to the nucleus upon activation, nuclear levels of NF-κB following bryostatin treatment (100 ng/ml) of NHA cells were measured using Western blotting and confocal microscopy. Increased nuclear levels of p65 could be detected after treatment of the cells with bryostatin for 10 min (Fig. 3b).

Moreover, confocal immunofluorescence microscopy was used to quantify the nuclear translocation of NF-κB p65 after bryostatin treatment at the indicated times (Fig. 3c, left). After single-cell measurements, an increase in p65 translocation was observed and quantified confirming the previous data (Fig. 3c, right). IKK-mediated phosphorylation of cytoplasmic IκB family members results in the proteasome-mediated degradation of IkB. We next examined IκBα protein levels in U-87 cells stimulated with bryostatin (Fig. 3d). We observed an increased rate of IκB degradation upon 5 min of bryostatin treatment.

Summing up, these data indicated that NF-κB was involved in activation of bryostatin-mediated viral reactivation in human astrocytes and primary cells.

### Activation of transcription of the HIV-1 LTR

**Figure 4:**
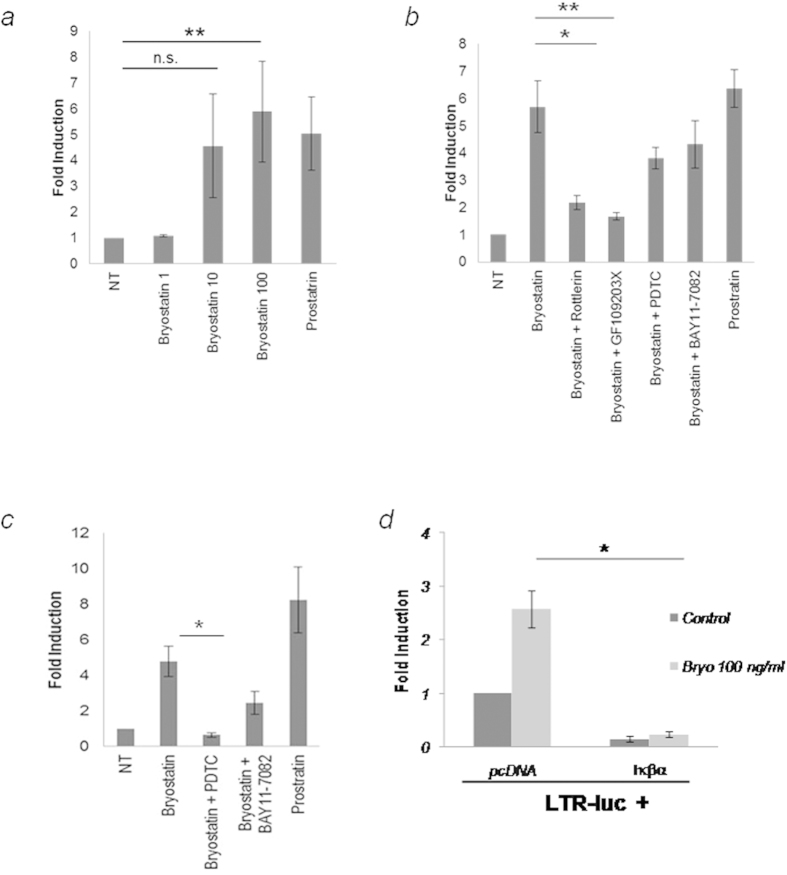
Induction of HIV-1 LTR activation by bryostatin. (**a**) U-87 cells (2.5 × 10^6^) were transiently transfected with pLTR-Luc (0.5 μg/10^6^ cells), and stimulated with different doses of bryostatin and prostratin (10 μg/ml). The luciferase activity in the cell lysates was measured 24 h later. Data represent the fold induction relative to non-treated cells (mean ± S.D. of four experiments performed in triplicate; **p < 0.01). (**b**) U-87 cells (2.5 × 10^6^) were transiently transfected with pLTR-Luc, treated with or without bryostatin alone or in combination with PKC and NF-κB inhibitors, and assayed for luciferase activity. Data represent the fold induction relative to bryostatin treated cells (mean ± S.D. of five experiments; *p ≤ 0.05, and **p < 0.01). (**c**) U-87 cells were transfected with the reporter κB plasmid, stimulated with bryostatin (100 ng/ml) alone or with NF-κB inhibitors, and assayed for luciferase activity. Data represent the fold induction relative to bryostatin-treated cells (mean ± S.D. of four experiments performed in triplicate; *p ≤ 0.05). (**d**) U-87 cells were co-transfected with the empty vector control, or IκBα, along with the pLTR-Luc reporter construct. Data represent the fold induction relative to bryostatin-treated cells transfected with the pcDNA (mean ± S.D. of three experiments performed in triplicate; *p ≤ 0.05).

Since the majority of transfection methods cause significant toxicity in primary cell cultures, all the experiments about the transcriptional activity of the LTR have been performed only in astrocytoma U-87 cells. Thus, to further study whether latent HIV-1 reactivation by bryostatin was a direct effect of LTR-transactivation, U-87 cells were transfected with a pLTR-Luc construct containing a 3′ LTR fragment of the HIV-1 genome that drives luciferase expression upon the transactivation of HIV-1 Tat^39^. Stimulation of U-87 cells with bryostatin induced a 3-fold increase in HIV-1 LTR-dependent luciferase activity in a dose-dependent manner (Fig. 4a). As expected, prostratin, which served as a positive control for NF-κB activation^40^ significantly increased LTR transcriptional activity.

To examine the impact of the different inhibitors that were used in our study on the effect of bryostatin, transfected LTRs were assayed for their responsiveness to bryostatin alone or in combination with PKC and NF-κB inhibitors. As expected, PKC inhibitors decreased the promoter activity to the control levels, and the addition of GF109203X reduced the effect of bryostatin by approximately 50%. Similarly, rottlerin inhibited LTR transcription by approximately 30% (Fig. 4b) and the use of the antioxidant PDTC reduced bryostatin induced LTR activation. Moreover, BAY 11–7082 inhibits NF-κB activation in around 20% compared with the treatment of bryostatin alone. As before, these results indicate that classic and novel PKCs, as well as the NF-κB transcription factor, are important for HIV-1 reactivation in human astrocytes.

To assess the functional role of NF-κB in bryostatin induced HIV-1 LTR activation, U-87 cells were transiently transfected with a pNF-κB-dependent luciferase reporter plasmid followed by bryostatin treatment alone or in combination with PDTC or BAY 11-7082 to verify the specificity of the signal. Bryostatin caused a marked, approximately 5-fold average increase in NF-κB-activity compared with the control, and as expected, this effect was abrogated by the two inhibitors tested, PDTC and BAY11-7082 (80% and 50%, respectively), thus confirming the specificity of the signal (Fig. 4c).

To dissect the precise involvement of NF-κB in the effects of bryostatin on HIV-1 LTR induction, U-87 cells were transiently co-transfected with the prototypical IκB member, IκBα, which was previously described to mediate NF-κB inhibition^41^ and the pLTR-Luc construct. As evidenced by the difference in luciferase activity, the only overexpression of IκBα was able to dramatically decrease the activity of the LTR promoter (Fig. 4d) and treatment with bryostatin did not modify this activation. These data are consistent with the cellular role of IκBα and further suggest that bryostatin-mediated induction of HIV-1 LTR in U-87 cells occurs through NF-κB activation.

### TNF-α could be a mediator of HIV-1 reactivation by bryostatin in human astrocytes

**Figure 5:**
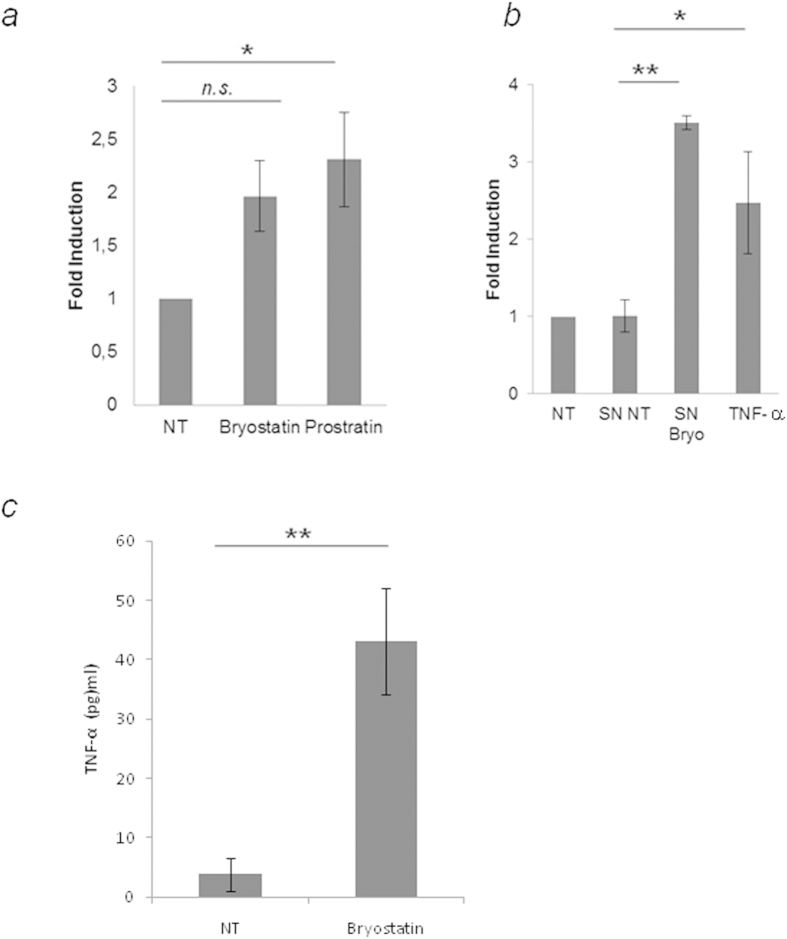
TNF-α could be a mediator of bryostatin mediated HIV-1 reactivation in human astrocytes. (**a**) U-87 cells (2.5 × 10^6^) were transiently transfected with pTNF-Luc, cultured in the presence of bryostatin (100 ng/ml) or prostratin (10 μg/ml) for 16 h, and assayed for luciferase activity (mean ± S.D. of three experiments performed in triplicate; *p ≤ 0.05). (**b**) U-87 cells (2.5 × 10^6^) transiently transfected with pLTR-Luc were treated with TNF-α and with supernatants from U-87 cells pretreated with bryostatin (100 ng/ml) during 16 h and assayed for luciferase activity (mean ± S.D. of three experiments performed in triplicate; (mean ± S.D. of three experiments performed in triplicate; *p ≤ 0.05, **p < 0.01). (**c**) Levels of TNF-α in the supernatants of U-87 cells treated during 24 h with bryostatin were measured by ELISA. The mean values (mean ± S.D.) of three independent experiments are shown (**p < 0.01).

It has been described that NF-κB activation by SDF-1α is indirectly mediated by TNF-α, such that TNF-α protein synthesis and secretion are up-regulated as a result of SDF-1α activation of MAPK in primary astrocytes^42^. Therefore, we verified in our system the possibility that bryostatin treatment induces the production of TNF-α, and that the newly synthesized and secreted TNF-α activates NF-κB signaling in an autocrine-dependent manner, initiating a second sequence of intracellular signaling events that could activate the LTR. To validate whether astrocytes induced HIV-1 reactivation occurred through TNF-α, U-87 cells were transiently transfected with a TNF-α promoter reporter plasmid, and then stimulated with bryostatin. Bryostatin was able to increase the transcriptional activity of the TNF-α promoter at similar levels as prostratin (Fig. 5a).

Moreover, we evaluated the effect of the supernatants of bryostatin treated cells on the activation of the LTR. We found strong LTR-luc activation in response to the supernatants of treated cells supporting the idea that bryostatin treatment induces the production of some mediators responsible for LTR induction and the subsequent HIV-1 activation (Fig. 5b). We do not discard that TNF-α is one of these mediators.

To confirm the involvement of TNF-α on effects mediated by bryostatin, U-87 cells were incubated with bryostatin for 24 h and supernatants examined by ELISA. The results show that production of TNF-α is upregulated in stimulated cells over controls (Fig. 5c).

## Discussion

Key research priorities for HIV-1 eradication include identifying anatomical and cellular reservoirs to develop targeted strategies that eliminate the virus from these sites^43^. In the brain, HIV-1 establishes latent or active infection primarily in astrocytes and microglia cells, where viral proteins are produced and shed^6^. Although the percentage of infected astrocytes *in vivo* is relatively low (approximately 2.6%), they are the most abundant cell type in the brain (approximately 0.4–2.0 × 10^12^ cells); hence, numerically, they may represent a significant source of viral persistence^1^. When stimulated with proinflammatory cytokines or when co-cultured with CD4+ cells, infected astrocytes release infectious HIV-1^44^,^45^, suggesting that when given the appropriate stimuli *in vivo*, astrocytes may serve as an on-going supply of HIV-1 in the brain.

The combination of HAART with new therapeutic agents that can reactivate latent reservoirs leads to HIV-1 eradication. In this regard, a non-tumor-promoting PKC inducer, bryostatin, has been shown to activate the MAPKs and NF-κB pathways and to synergize with HDACi to reactivate HIV-1 gene expression in latently infected J-Lat cell lines^46^. Interestingly, a recent study^47^ has shown that nanoparticles loaded with bryostatin target and activate primary human CD4+ T cells and stimulate latent virus production *in vitro* from latently infected J-Lat 8.4 and 10.6 cell line, and from latently infected cells in a humanized mouse model, SCID-hu *ex vivo*.

Astrocyte cultures exposed to HIV-1 show initial transient virus production in a subpopulation of cells and virus production peaks at approximately day 2-7 post infection^7^. After the initial acute infection period, HIV-1 production decreases to very low or undetectable levels and persistent HIV-1 infection is established. We demonstrated for the first time that bryostatin is potently active in the reactivation of HIV-1-infected astrocytes, as previously described for TNF-α and IL-1β^45^,^48^.

It is also known that bryostatin-1 induces the proliferation and activation of B and T cells^49^. Importantly, we ruled out that the effects on HIV-1 reactivation are due to changes in proliferation activity because no changes in Ki-67-positive cells were found after bryostatin treatment. Moreover, when the levels of GFAP protein, marker for astrogliosis, were analyzed after treatment, we did not find any modification of basal expression of GFAP (unpublished results).

PKCs are classified into three subfamilies: conventional or classical (α, βI, βII, and γ), novel (δ, ε, η, and θ), and atypical (λ, and ξ), depending on their subcellular localization, biochemical properties, and substrate specificity^23^. We attempted to decipher which of the isoenzymes are involved in the latent HIV-1 reactivation process by using specific inhibitors of classical and novel isoenzymes. After pretreatment with GF109203X and rottlerin, we found that both were able to ablate the bryostatin induced HIV-1-reactivation in U-87 cells, and more importantly, in primary cells.

Bryostatin induces PKC, which is responsible for a wide range of activities such as cellular proliferation and NF-κB activation. The main effect of NF-κB on the bryostatin effects was confirmed by using PDTC in viral reactivation experiments, which showed that bryostatin-mediated HIV-1 reactivation was abrogated. Under these conditions, bryostatin also induced NF-κB nuclear translocation, resulting in transcription. The HIV-1 LTR is regulated, in large part, by cellular transcription factors, including NF-κB, AP-1, SP-1, and NFAT^50^. Consequently, stimuli that activate these transcription factors cause HIV-1 LTR-driven viral replication^51^. Here, we report a dose-dependent activation of the HIV-1 LTR by bryostatin in U-87 cells transfected with an LTR-containing vector. The involvement of classical and novel PKCs was confirmed in transfection assays by using GF109203X and rottlerin. As expected, both drugs prevented the HIV-1 reactivation process due to bryostatin. In addition to NF-κB, PKC induces HIV-1 LTR transcription via activation of the AP-1 transcription factor; however, its action is largely dependent upon cooperation with NF-κB^52^,^53^. We are presently researching the roles of AP-1 and NFAT at the HIV-1 promoter that lead to virus reactivation after bryostatin treatment in human astrocytes.

It has been previously described that PKC agonists down-regulate the expression of the HIV-1 CD4 receptor and the CXCR4 and CCR5 coreceptors on the host cell surface^54^,^55^,^56^. Although human astrocytes are CD4-negative, we ruled out any effect of bryostatin on HIV-1 coreceptors in these cells. Preincubation of primary astrocytes with bryostatin had no significant effect on the expression of CXCR4 or CCR5 (data not shown). Moreover, in accordance with this result, when NHA cells were pretreated with variable concentrations of bryostatin, we did not observe any effect on HIV-1 replication when compared with non-treated infected cells (unpublished results).

It has been previously reported that co-administration of paclitaxel and bryostatin 1 results in potentiation of TNF-α mRNA levels and prolonged protein accumulation in U937 cells^57^. Because the main role of this cytokine is clearly as a potent inductor of HIV-1 replication, we investigated its role as a mediator of bryostatin effects. Although preliminary, our results show increased the transcriptional activity of the TNF-α promoter in U-87 cells after bryostatin stimulation. Future experiments will be conducted to confirm this hypothesis.

In conclusion, we show that bryostatin can directly increase HIV-1 LTR activity in human astrocytes (primary and astrocytoma cells) *in vitro* via the PKC pathway and an NF-κB-dependent mechanism. Therefore, it is plausible that HIV-1-infected astrocytes exposed to bryostatin may contribute to HIV-1 latency activation and will provide a foundation for future novel HIV-1-purging strategies from tissue reservoirs such as the CNS.

## Methods

### Cell culture and treatments

Normal human astrocytes (NHA) isolated from the cerebrums of 5-month-old human fetuses were purchased from Cambrex (CC-2565, Walkersville, MD, USA), and cultured according to the manufacturer’s protocol. The astrocytoma human cell line U-87 was routinely grown in cultured in Dulbecco’s modified Eagle’s medium (DMEM) (Gibco, Rockville, MD, USA) containing 10% heat-inactivated fetal calf serum, 1% penicillin/streptomycin, and 2 mM L-glutamine (ICN Pharmaceuticals, CA, USA) at 37 °C in a humidified atmosphere of 5% CO_2_.

Bryostatin-1, prostratin, GF109203X, and rottlerin were purchased from Sigma (St. Louis, MO, USA). Pyrrolidine dithiocarbamate (PDTC), and BAY11-7082 were obtained from Santa Cruz Biotechnology (Santa Cruz CA, USA).

### HIV-1 viral isolates

Virus stocks were prepared by amplifying of X4 HIV-1_NL4.3_ virus in MT-2 cells (ATCC). HIV-1 viral stocks were evaluated by quantifying HIV p24gag using an enzyme-linked immunosorbent assay (ELISA) (Innotest HIV-1 antigen mAb; Innogenetic, Ghent, Belgium).

### HIV-1 viral reactivation

NHA cells were infected for 2 h at a concentration of 100 ng p24/10^6^ of cells. After washing, infected cells were kept in culture, and the p24 concentration was measured in the culture supernatants using an ELISA (Innotest HIV-1 antigen mAb; Innogenetic, Ghent, Belgium).

### Plasmid Constructs

The reporter pNF-κB-Luc expression vector contains 3 tandem copies of the NF-κB site of the conalbumin promoter driving the luciferase reporter gene. The IκBα expression plasmid was also generously provided by Dr. G. Crabtree and the reporter plasmid pLTR-Luc was a gift from Dr J. L. Virelizier (Institute Pasteur, Paris, France). The pcDNA3 plasmid (Invitrogen, Carlsbad, CA, USA) which is a cloning vector containing the CMV promoter was used in our experiments as a control for the transfection in expression plasmids or to adjust the quantities of transfected DNA. The human TNF-α promoter was provided by Dr. J. Economou (UCLA School of Medicine, Los Angeles, CA)^58^.

### Transfection and Luciferase Assays

U-87 cells were transiently transfected by using Lipofectamine Plus Reagent (Invitrogen Life Technologies) following the manufacturer’s instructions. The protein content was measured using the bicinchoninic acid method (Pierce) according to the manufacturer’s instructions and luciferase activity was determined as the ratio of firefly to *Renilla* luciferase using the Luminometer 1450 Microbeta Luminiscence Counter.

### Western blotting

NHA and U-87 cells were exposed to bryostatin, washed with phosphate buffered saline (PBS) and lysed with lysis buffer. Protein contents were measured using bicinchoninic acid protein assay. For western blotting, 30 μg of protein from each sample was subjected to sodium dodecyl sulfate-polyacrylamide gel electrophoresis on a 7.5% gel and transferred to a polyvinylidene fluoride membrane (Millipore, Bedford, MA, USA) by semidry transference blotting. Membranes were blocked in 5% milk for 1 h, washed with Tris-buffered saline with 0.05% Tween 20, and incubated with the primary antibody for 2 h. Immunoblotting was conducted by using either anti-p65 active subunit MAB3026 (Millipore, Bedford, MA, USA.), a mouse anti-IκBα subunit (Santa Cruz Biotechnology, Inc), anti-tubulin or anti-NuMA (Sigma, St. Louis, MO) antibodies at a 1:1000 dilution. Immunoreactive bands were detected by incubating the blot with secondary antibodies conjugated to peroxidase for 1 h (Amersham-Pharmacia Biotech; 1:5000). Proteins were detected using the enhanced chemiluminescence system (Amersham-Pharmacia Biotech). To detect NF-κB nuclear translocation, nuclear and cytosolic protein extracts were obtained using a Nuclear/Cytosolic fractionation kit (MBL International).

### TNF-α detection in cultured supernatants by ELISA

U-87 cells (24-well plate, 5 10^5^ cells/well) were incubated with Bryostatin (100 ng/ml) for 24 h at 37 °C in humidified 5% CO_2_. Culture supernatants were harvested and centrifuged to remove cellular debris, and aliquots are stored at –80 °C until assayed. Specific immunoreactivity to TNF-α (Thermo Scientific, Pierce) was measured by ELISA according to the manufacturers’ instructions. The sensitivity of the assay was 2 pg/mL.

### Confocal analysis

Cells were fixed in PBS (pH 7.4) with 3.7% paraformaldehyde and 0.025% glutaraldehyde for 10 min. Fixed cells were permeabilized in PBS with 0.1% Triton for 10 min. After two washes, the cells were incubated with 1% PBS-BSA (pH 7.4) for 20 min, and immunofluorescence staining was performed using a rabbit anti-human p65/relA antibody (AB 1604a) (dilution 1/500-1/1000) (Millipore, Bedford, MA, USA), followed by incubation with a secondary antibody conjugated to FITC. TO-PRO®-3 iodide (642/661) (Life Technologies) was applied to label the nuclei and confocal laser scanning microscopy was performed using a LEICA AOBS-TCS-SP2 system. Separate images were taken in the corresponding channels, and merged images were composed. Image acquisition and data processing for all samples were performed under the same conditions.

### Cell proliferation

Cell proliferation was measured using intracellular staining with Ki-67 (BD Pharmingen), as indicated by the manufacturer. DMEM 10% was used as positive control of U-87 proliferation.

### MTT assay

MTT assay measures the reduction of a tetrazolium component (MTT) into an insoluble formazan product by the mitochondria of viable cells. Briefly, cells were treated with bryostatin and after 24, 48, and 72 h 20 μl MTT (3-(4,5-dimethylthiazol-2-yl)-2,5-diphenyl-tetrazolium-bromide) substrate solution (5 mg/ml) was added to the cells to measure mitochondrial activity. After 4 h, the supernatant was removed, and formazan crystals were dissolved in 200 μl dimethyl sulfoxide (DMSO) (Sigma, St. Louis, MO, USA). All points were performed in triplicate. DMSO 20% was used as positive control of toxicity.

### Statistical analysis

The data are expressed as the mean ± S.D. of three to five independent experiments. Differences were analyzed using non-parametric tests (Mann–Whitney “U”) and were considered significant when *p ≤ 0.05 and **p < 0.01.

## Additional Information

**How to cite this article**: Díaz, L. *et al.* Bryostatin activates HIV-1 latent expression in human astrocytes through a PKC and NF-KB-dependent mechanism. *Sci. Rep.* **5**, 12442; doi: 10.1038/srep12442 (2015).

## Supplementary Material

Supplementary Information

## References

[b1] Brack-Werner R. Astrocytes: HIV cellular reservoirs and important participants in neuropathogenesis. AIDS 13, 1–22 (1999). 10207540 10.1097/00002030-199901140-00003

[b2] Canki M. *et al.* Highly productive infection with pseudotyped human immunodeficiency virus type 1 (HIV-1) indicates no intracellular restrictions to HIV-1 replication in primary human astrocytes. J Virol 75, 7925–7933 (2001). 11483737 10.1128/JVI.75.17.7925-7933.2001 PMC115036

[b3] Clarke J. N. *et al.* Novel pathway of human immunodeficiency virus type 1 uptake and release in astrocytes. Virology 348, (2006). 10.1016/j.virol.2005.12.004 16445956

[b4] Speth C., Dierich M. P. & Sopper S. HIV-infection of the central nervous system: the tightrope walk of innate immunity. Mol Immunol 42, 213–228 (2005). 15488609 10.1016/j.molimm.2004.06.018

[b5] Gonzalez-Scarano F. & Martin-Garcia J. The neuropathogenesis of AIDS. Nat Rev Immunol 5, 69–81 (2005). 15630430 10.1038/nri1527

[b6] Kaul M., Garden G. A. & Lipton S. A. Pathways to neuronal injury and apoptosis in HIV-associated dementia. Nature 410, 988–994 (2001). 11309629 10.1038/35073667

[b7] Kramer-Hammerle S., Rothenaigner I., Wolff H., Bell J. E. & Brack-Werner R. Cells of the central nervous system as targets and reservoirs of the human immunodeficiency virus. Virus Res 111, 194–213 (2005). 15885841 10.1016/j.virusres.2005.04.009

[b8] Minagar A. *et al.* The role of macrophage/microglia and astrocytes in the pathogenesis of three neurologic disorders: HIV-associated dementia, Alzheimer disease, and multiple sclerosis. J Neurol Sci 202, 13–23 (2002). 12220687 10.1016/s0022-510x(02)00207-1

[b9] Schnell G., Price R. W., Swanstrom R. & Spudich S. Compartmentalization and clonal amplification of HIV-1 variants in the cerebrospinal fluid during primary infection. J Virol 84, 2395–2407 (2010). 20015984 10.1128/JVI.01863-09 PMC2820937

[b10] Schnell G., Joseph S., Spudich S., Price R. W. & Swanstrom R. HIV-1 replication in the central nervous system occurs in two distinct cell types. PLoS Pathog 7, e1002286 (2011). 22007152 10.1371/journal.ppat.1002286 PMC3188520

[b11] Lehrman G. *et al.* Depletion of latent HIV-1 infection *in vivo*: a proof-of-concept study. Lancet 366, 549–555 (2005). 16099290 10.1016/S0140-6736(05)67098-5 PMC1894952

[b12] Lindkvist A. *et al.* Reduction of the HIV-1 reservoir in resting CD4+ T-lymphocytes by high dosage intravenous immunoglobulin treatment: a proof-of-concept study. AIDS Res Ther 6, 15 (2009). 19570221 10.1186/1742-6405-6-15 PMC2713257

[b13] Prins J. M. *et al.* Immuno-activation with anti-CD3 and recombinant human IL-2 in HIV-1-infected patients on potent antiretroviral therapy. AIDS 13, 2405–2410 (1999). 10597782 10.1097/00002030-199912030-00012

[b14] Bisgrove D., Lewinski M., Bushman F. & Verdin E. Molecular mechanisms of HIV-1 proviral latency. Expert Rev Anti Infect Ther 3, 805–814 (2005). 16207172 10.1586/14787210.3.5.805

[b15] Han Y., Wind-Rotolo M., Yang H. C., Siliciano J. D. & Siliciano R. F. Experimental approaches to the study of HIV-1 latency. Nat Rev Microbiol 5, 95–106 (2007). 17224919 10.1038/nrmicro1580

[b16] Scripture-Adams D. D., Brooks D. G., Korin Y. D. & Zack J. A. Interleukin-7 induces expression of latent human immunodeficiency virus type 1 with minimal effects on T-cell phenotype. J Virol 76, 13077–13082 (2002). 12438635 10.1128/JVI.76.24.13077-13082.2002 PMC136703

[b17] van Praag R. M. *et al.* OKT3 and IL-2 treatment for purging of the latent HIV-1 reservoir *in vivo* results in selective long-lasting CD4+ T cell depletion. J Clin Immunol 21, 218–226 (2001). 11403229 10.1023/a:1011091300321

[b18] Davidson S. K., Allen S. W., Lim G. E., Anderson C. M. & Haygood M. G. Evidence for the biosynthesis of bryostatins by the bacterial symbiont “Candidatus Endobugula sertula” of the bryozoan Bugula neritina. Appl Environ Microbiol 67, 4531–4537 (2001). 11571152 10.1128/AEM.67.10.4531-4537.2001 PMC93199

[b19] Newton A. C. Protein kinase C: poised to signal. Am J Physiol Endocrinol Metab 298, E395–402 (2010). 19934406 10.1152/ajpendo.00477.2009 PMC2838521

[b20] Reyland M. E. Protein kinase C isoforms: Multi-functional regulators of cell life and death. Front Biosci (Landmark Ed) 14, 2386–2399 (2009). 19273207 10.2741/3385 PMC5204454

[b21] He X. *et al.* Bryostatin-5 blocks stromal cell-derived factor-1 induced chemotaxis via desensitization and down-regulation of cell surface CXCR4 receptors. Cancer Res 68, 8678–8686 (2008). 18974109 10.1158/0008-5472.CAN-08-0294

[b22] Boto W. M., Brown L., Chrest J. & Adler W. H. Distinct modulatory effects of bryostatin 1 and staurosporine on the biosynthesis and expression of the HIV receptor protein (CD4) by T cells. Cell Regul 2, 95–103 (1991). 1863603 10.1091/mbc.2.2.95 PMC361724

[b23] Mehla R. *et al.* Bryostatin modulates latent HIV-1 infection via PKC and AMPK signaling but inhibits acute infection in a receptor independent manner. PLoS One 5, e11160 (2010). 20585398 10.1371/journal.pone.0011160 PMC2886842

[b24] Asiedu C., Biggs J., Lilly M. & Kraft A. S. Inhibition of leukemic cell growth by the protein kinase C activator bryostatin 1 correlates with the dephosphorylation of cyclin-dependent kinase 2. Cancer Res 55, 3716–3720 (1995). 7641182

[b25] Nekhai S. *et al.* HIV-1 Tat-associated RNA polymerase C-terminal domain kinase, CDK2, phosphorylates CDK7 and stimulates Tat-mediated transcription. Biochem J 364, 649–657 (2002). 12049628 10.1042/BJ20011191 PMC1222613

[b26] Vlach J. & Pitha P. M. Activation of human immunodeficiency virus type 1 provirus in T-cells and macrophages is associated with induction of inducer-specific NF-kappa B binding proteins. Virology 187, 63–72 (1992). 1371030 10.1016/0042-6822(92)90295-z

[b27] Mutter R. & Wills M. Chemistry and clinical biology of the bryostatins. Bioorg Med Chem 8, 1841–1860 (2000). 11003129 10.1016/s0968-0896(00)00150-4

[b28] Propper D. J. *et al.* A phase II study of bryostatin 1 in metastatic malignant melanoma. Br J Cancer 78, 1337–1341 (1998). 9823975 10.1038/bjc.1998.680 PMC2063191

[b29] Varterasian M. L. *et al.* Phase II trial of bryostatin 1 in patients with relapsed low-grade non-Hodgkin’s lymphoma and chronic lymphocytic leukemia. Clin Cancer Res 6, 825–828 (2000). 10741703

[b30] Varterasian M. L. *et al.* Phase I study of bryostatin 1 in patients with relapsed non-Hodgkin’s lymphoma and chronic lymphocytic leukemia. J Clin Oncol 16, 56–62 (1998). 9440723 10.1200/JCO.1998.16.1.56

[b31] Varterasian M. L. *et al.* Phase II study of bryostatin 1 in patients with relapsed multiple myeloma. Invest New Drugs 19, 245–247 (2001). 11561682 10.1023/a:1010676719178

[b32] Mohammad R. M. *et al.* Sequential treatment of human chronic lymphocytic leukemia with bryostatin 1 followed by 2-chlorodeoxyadenosine: preclinical studies. Clin Cancer Res 4, 445–453 (1998). 9516935

[b33] Etcheberrigaray R. *et al.* Therapeutic effects of PKC activators in Alzheimer’s disease transgenic mice. Proc Natl Acad Sci USA 101, 11141–11146 (2004). 15263077 10.1073/pnas.0403921101 PMC503753

[b34] Nelson T. J., Sen A., Alkon D. L. & Sun M. K. Adduct formation in liquid chromatography-triple quadrupole mass spectrometric measurement of bryostatin 1. J Chromatogr B Analyt Technol Biomed Life Sci 944, 55–62 (2014). 10.1016/j.jchromb.2013.11.020 24291721

[b35] Reynolds J. L. *et al.* Proteomic analysis of the effects of cocaine on the enhancement of HIV-1 replication in normal human astrocytes (NHA). Brain Res 1123, 226–236 (2006). 17034766 10.1016/j.brainres.2006.09.034 PMC1751122

[b36] Folgueira L. *et al.* Protein kinase C-zeta mediates NF-kappa B activation in human immunodeficiency virus-infected monocytes. J Virol 70, 223–231 (1996). 8523529 10.1128/jvi.70.1.223-231.1996 PMC189808

[b37] Alvarez S., Serramia M. J., Fresno M. & Munoz-Fernandez M. A. HIV-1 envelope glycoprotein 120 induces cyclooxygenase-2 expression in astrocytoma cells through a nuclear factor-kappaB-dependent mechanism. Neuromolecular Med 9, 179–193 (2007). 17627037 10.1007/BF02685891

[b38] Alvarez S., Blanco A., Kern F., Fresno M. & Munoz-Fernandez M. A. HIV-2 induces NF-kappaB activation and cyclooxygenase-2 expression in human astroglial cells. Virology 380, 144–151 (2008). 18752821 10.1016/j.virol.2008.07.008

[b39] Jeeninga R. E. *et al.* Functional differences between the long terminal repeat transcriptional promoters of human immunodeficiency virus type 1 subtypes A through G. J Virol 74, 3740–3751 (2000). 10729149 10.1128/jvi.74.8.3740-3751.2000 PMC111883

[b40] Kulkosky J. *et al.* Expression of latent HAART-persistent HIV type 1 induced by novel cellular activating agents. AIDS Res Hum Retroviruses 20, 497–505 (2004). 15186524 10.1089/088922204323087741

[b41] Ghosh S., May M. J. & Kopp E. B. NF-kappa B and Rel proteins: evolutionarily conserved mediators of immune responses. Annu Rev Immunol 16, 225–260 (1998). 9597130 10.1146/annurev.immunol.16.1.225

[b42] Han Y., He T., Huang D. R., Pardo C. A. & Ransohoff R. M. TNF-alpha mediates SDF-1 alpha-induced NF-kappa B activation and cytotoxic effects in primary astrocytes. J Clin Invest 108, 425–435 (2001). 11489936 10.1172/JCI12629 PMC209361

[b43] Thomas C. Roadblocks in HIV research: five questions. Nat Med 15, 855–859 (2009). 19661992 10.1038/nm0809-855

[b44] Sabri F. *et al.* Nonproductive human immunodeficiency virus type 1 infection of human fetal astrocytes: independence from CD4 and major chemokine receptors. Virology 264, 370–384 (1999). 10562499 10.1006/viro.1999.9998

[b45] Tornatore C., Nath A., Amemiya K. & Major E. O. Persistent human immunodeficiency virus type 1 infection in human fetal glial cells reactivated by T-cell factor(s) or by the cytokines tumor necrosis factor alpha and interleukin-1 beta. J Virol 65, 6094–6100 (1991). 1920627 10.1128/jvi.65.11.6094-6100.1991 PMC250285

[b46] Perez M. *et al.* Bryostatin-1 synergizes with histone deacetylase inhibitors to reactivate HIV-1 from latency. Curr HIV Res 8, 418–429 (2010). 20636281 10.2174/157016210793499312

[b47] Kovochich M., Marsden M. D. & Zack J. A. Activation of latent HIV using drug-loaded nanoparticles. PLoS One 6, e18270 (2011). 21483687 10.1371/journal.pone.0018270 PMC3071729

[b48] Tornatore C., Meyers K., Atwood W., Conant K. & Major E. Temporal patterns of human immunodeficiency virus type 1 transcripts in human fetal astrocytes. J Virol 68, 93–102 (1994). 8254781 10.1128/jvi.68.1.93-102.1994 PMC236268

[b49] Scheid C. *et al.* Immunomodulation in patients receiving intravenous Bryostatin 1 in a phase I clinical study: comparison with effects of Bryostatin 1 on lymphocyte function *in vitro*. Cancer Immunol Immunother 39, 223–230 (1994). 7954524 10.1007/BF01525985 PMC11038930

[b50] Jones K. A. & Peterlin B. M. Control of RNA initiation and elongation at the HIV-1 promoter. Annu Rev Biochem 63, 717–743 (1994). 7979253 10.1146/annurev.bi.63.070194.003441

[b51] Duh E. J., Maury W. J., Folks T. M., Fauci A. S. & Rabson A. B. Tumor necrosis factor alpha activates human immunodeficiency virus type 1 through induction of nuclear factor binding to the NF-kappa B sites in the long terminal repeat. Proc Natl Acad Sci USA 86, 5974–5978 (1989). 2762307 10.1073/pnas.86.15.5974 PMC297754

[b52] Roebuck K. A., Gu D. S. & Kagnoff M. F. Activating protein-1 cooperates with phorbol ester activation signals to increase HIV-1 expression. AIDS 10, 819–826 (1996). 8828738 10.1097/00002030-199607000-00004

[b53] Yang X., Chen Y. & Gabuzda D. ERK MAP kinase links cytokine signals to activation of latent HIV-1 infection by stimulating a cooperative interaction of AP-1 and NF-kappaB. J Biol Chem 274, 27981–27988 (1999). 10488148 10.1074/jbc.274.39.27981

[b54] Warrilow D., Gardner J., Darnell G. A., Suhrbier A. & Harrich D. HIV type 1 inhibition by protein kinase C modulatory compounds. AIDS Res Hum Retroviruses 22, 854–864 (2006). 16989610 10.1089/aid.2006.22.854

[b55] Gulakowski R. J., McMahon J. B., Buckheit R. W. Jr., Gustafson K. R. & Boyd M. R. Antireplicative and anticytopathic activities of prostratin, a non-tumor-promoting phorbol ester, against human immunodeficiency virus (HIV). Antiviral Res 33, 87–97 (1997). 9021050 10.1016/s0166-3542(96)01004-2

[b56] Hezareh M. *et al.* Mechanisms of HIV receptor and co-receptor down-regulation by prostratin: role of conventional and novel PKC isoforms. Antivir Chem Chemother 15, 207–222 (2004). 15457682 10.1177/095632020401500404

[b57] Wang S., Wang Z., Dent P. & Grant S. Induction of tumor necrosis factor by bryostatin 1 is involved in synergistic interactions with paclitaxel in human myeloid leukemia cells. Blood 101, 3648–3657 (2003). 12522001 10.1182/blood-2002-09-2739

[b58] Rhoades K. L., Cai S., Golub S. H. & Economou J. S. Granulocyte-macrophage colony-stimulating factor and interleukin-4 differentially regulate the human tumor necrosis factor-alpha promoter region. Cell Immunol 161, 125–131 (1995). 7867077 10.1006/cimm.1995.1016

