# Supplementary material for: Bryostatin activates HIV-1 latent expression in human astrocytes through a PKC and NF-ĸB-dependent mechanism
Source: Sci Rep. 2015 Jul 22;5:12442. doi: 10.1038/srep12442 (PMC4510492; doi:10.1038/srep12442)
Supplement: Supplementary Information [file srep12442-s1.pdf]

Supplementary Information

Title

Bryostatin activates HIV-1 latent expression in human astrocytes through a PKC and NF-κB-dependent mechanism

Runing Title: HIV-1 brain reactivation by Bryostatin

Authors: Laura Díaz,*[,1] Marta Martínez-Bonet,*[,1] Javier Sánchez,* Alejandra Fernández-Pineda,* José Luis Jiménez, Eduardo Muñoz,[§] Santiago Moreno,[#] Susana Álvarez,*[,&] and Mª Ángeles Muñoz-Fernández*[,&]

Supplemental Information

Figure S1. **Bryostatin is nontoxic at its active concentration.** a) NHA and b) U-87 cells were treated with various concentrations of bryostatin for 24, 48 and 72 h, and cell survival was determined using the MTT cytotoxicity assay. Each treatment was performed in triplicate. (n = 2).

Figure S2. **Bryostatin does not affect cell proliferation.** a) NHA and b) U-87 cells were treated with bryostatin (100 ng/ml) for the indicated times and cell proliferation was measured by staining with the anti-Ki-67 antibody. DMEM 10% was used as positive control of U-87 proliferation. The mean values (mean ± S.D.) of three independent experiments are shown.

Figure S3. NF-κB is involved in the bryostatin induced HIV-1 reactivation in astrocytoma cells. The p24 levels in U-87 cell supernatants were monitored 3, 6, 8, and 10 dpi after treatment with bryostatin alone or in combination with PDTC (10 μM), and two doses of BAY11-7082 (20 and 40 μM). The d10/d8 dpi ratio of U-87 cells is shown. The results of three independent experiments expressed as the fold increase relative to that of infected cells.

a

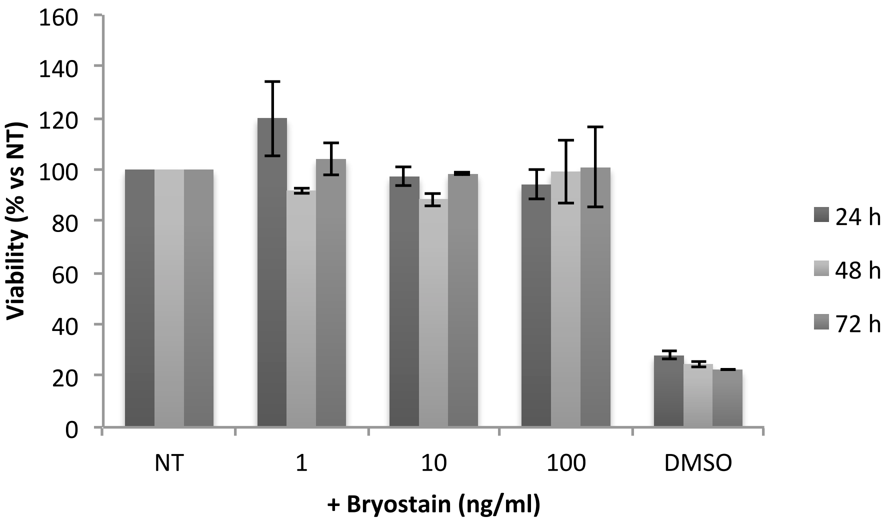

160
140
120
100
80
60
40
20
0
Viability (% vs NT)
NT
1
10
100
DMSO
+ Bryostain (ng/ml)
24 h
48 h
72 h

b

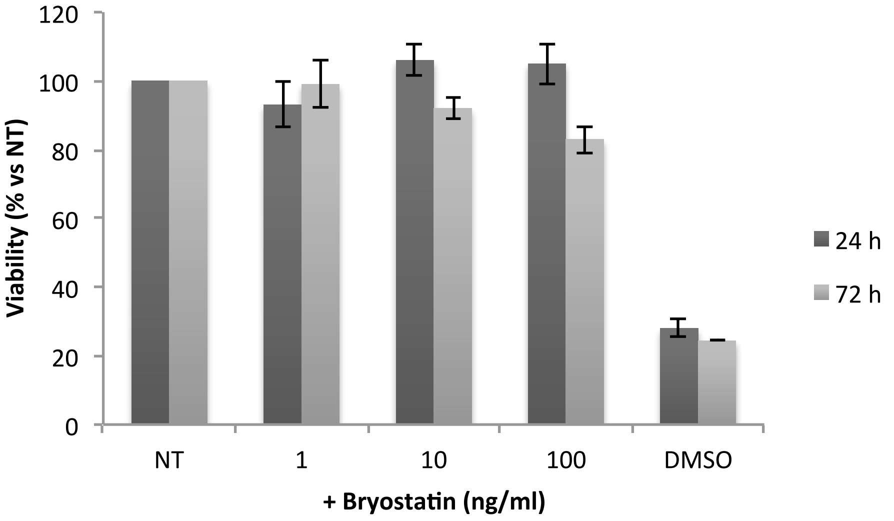

120
100
80
60
40
20
0
Viability (% vs NT)
NT
1
10
100
DMSO
+ Bryostatin (ng/ml)
24 h
72 h

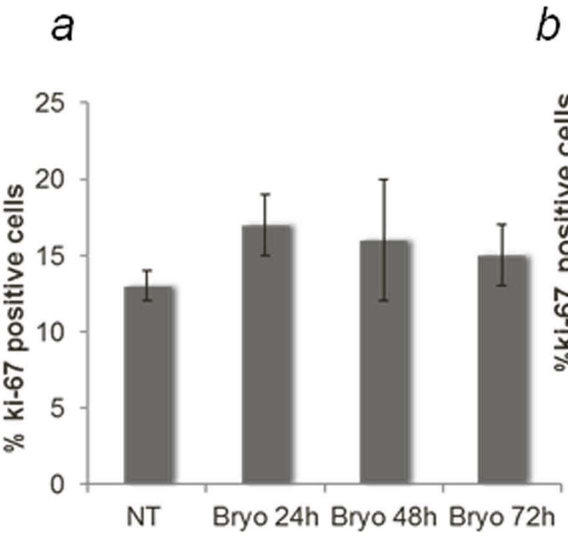

a
% ki-67 positive cells
25
20
15
10
5
0
NT
Bryo 24h
Bryo 48h
Bryo 72h

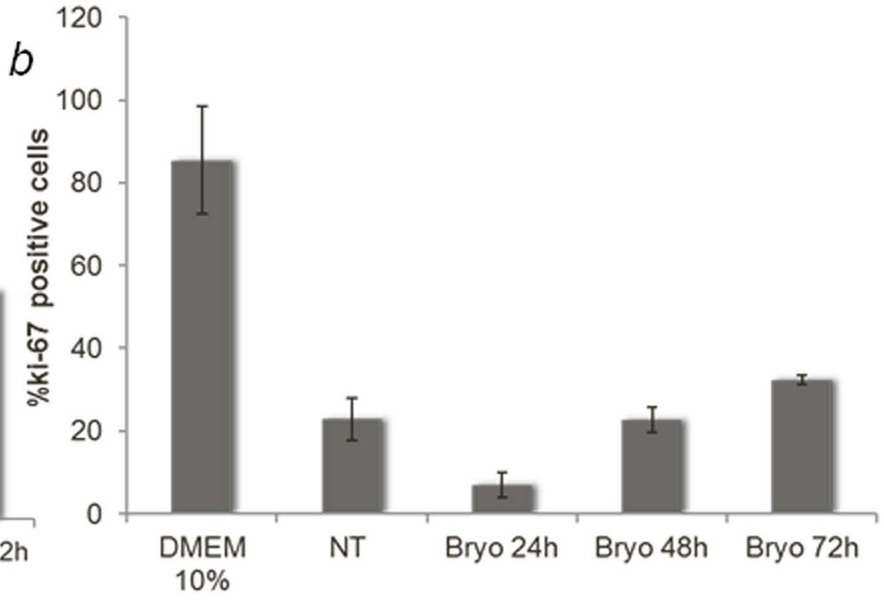

b
%ki-67 positive cells
120
100
80
60
40
20
0
DMEM 10%
NT
Bryo 24h
Bryo 48h
Bryo 72h

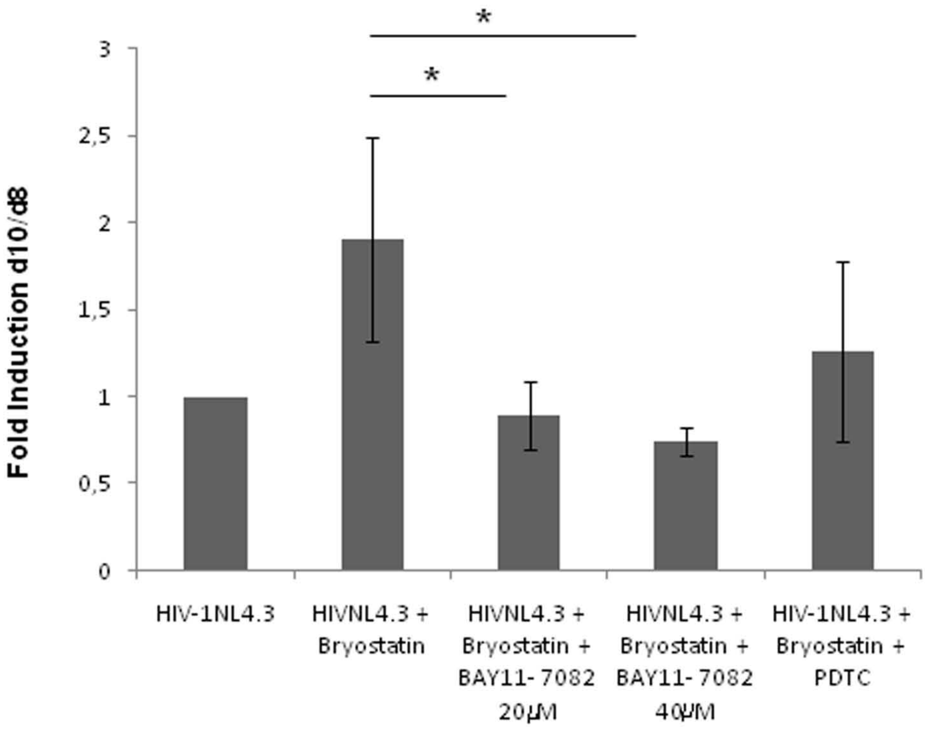
*
*
Fold Induction d10/d8
3
2,5
2
1,5
1
0,5
0
HIV-1NL4.3
HIVNL4.3 + Bryostatin
HIVNL4.3 + Bryostatin + BAY11- 7082 20µM
HIVNL4.3 + Bryostatin + BAY11- 7082 40µM
HIV-1NL4.3 + Bryostatin + PDTC
